# Tau Phosphorylation Rates Measured by Mass Spectrometry Differ in the Intracellular Brain vs. Extracellular Cerebrospinal Fluid Compartments and Are Differentially Affected by Alzheimer’s Disease

**DOI:** 10.3389/fnagi.2019.00121

**Published:** 2019-05-21

**Authors:** Nicolas R. Barthélemy, Nipun Mallipeddi, Paul Moiseyev, Chihiro Sato, Randall J. Bateman

**Affiliations:** ^1^Department of Neurology, Washington University School of Medicine, St. Louis, MO, United States; ^2^Hope Center for Neurological Disorders, Washington University School of Medicine, St. Louis, MO, United States; ^3^Charles F. and Joanne Knight Alzheimer’s Disease Research Center, Washington University School of Medicine, St. Louis, MO, United States

**Keywords:** tau, phosphorylation, brain, mass spectrometry-LC-MS/MS, cerebrospinalfluid, parallel reaction monitoring, quantification, Alzheimer’s disease

## Abstract

Tau protein aggregation into neurofibrillary tangles in the central nervous system contributes to the etiology of certain neurodegenerative disorders, including Alzheimer’s disease (AD). Though the mechanism of tau destabilization is not fully understood yet, tau protein has been found to be hyperphosphorylated in tau aggregates. To investigate this further, we developed a highly sensitive and specific mass spectrometry (MS) method using parallel reaction monitoring (PRM) to identify tau phosphorylation sites. This method enables us to compare the abundance of phosphorylation sites in tau proteins in the brain and cerebrospinal fluid (CSF) in humans with and without AD. We detected 29 distinct phosphorylated tau (p-tau) sites in full-length tau from soluble human brain lysate and 12 sites on truncated tau in CSF, mainly in the mid-domain. Brain soluble tau phosphorylation sites are localized on three domains including a proline-rich mid-domain, the C-terminus, and a cluster on the N-terminal projection domain not previously characterized. Some phosphorylation sites increased in CSF, while others decreased compared to brain. Notably, phosphorylation on T205 and S208, recognized by AT8 antibody defining Braak stages of brain tau aggregation, were not detected in normal brain soluble tau but were found in the CSF. Comparison of the p-tau rates from the brain and the CSF indicated that the abundance of phosphorylated sites varied in a site-specific manner. CSF tau proteins from non-AD participants were significantly hyperphosphorylated on T111, T205, S208, T217 and T231. In AD CSF, hyperphosphorylation on these sites was exacerbated, and phosphorylation on T153 and T175 specifically were detected. This supports the hypothesis that tau hyperphosphorylation could be a physiological process amplified by AD pathology. Conversely, we found that S202 was hypophosphorylated in CSF and was not hyperphosphorylated in AD, demonstrating that p-tau isoforms could have different metabolisms depending on which sites are phosphorylated. These site-specific p-tau rates are independent of tau concentration and distinct of current CSF tau and p-tau assays measuring tau isoforms levels. Targeted MS multiplexing ability and high-throughput capacity lets us envision the use of these new p-tau measurements as promising biomarkers for AD diagnosis and tracking therapeutic responses.

## Introduction

Tau protein aggregation in the brain is one of the hallmarks of neurodegenerative diseases called tauopathies, including Alzheimer disease (AD), some frontotemporal dementia (FTD), progressive supranuclear palsy (PSP), Pick’s disease, and corticobasal degeneration (CBD). Normally a soluble protein, there is a growing body of evidence associating insoluble tau accumulation in the brain with cognitive decline (Villemagne et al., 2015; Schöll et al., 2016). However, to date, the biological and molecular pathways leading to the abnormal tau proteins observed in the different tauopathies are not well understood. One of the main hypotheses regarding the origin of the pathogenesis of AD attributes abnormal tau phosphorylation patterns to its disassembly from microtubules and aggregation as paired helical filaments (PHF; Iqbal et al., 2005; Wang and Mandelkow, 2016). Supporting this hypothesis, abnormally-phosphorylated tau (p-tau) has been detected in AD tau aggregates using phospho-specific antibodies (Köpke et al., 1993; Braak and Braak, 1995; Augustinack et al., 2002). Despite evidence that tau phosphorylation can induce its self-assembly, the exact mechanism or the critical phosphorylation sites are a matter of debate (Alonso et al., 2001). In this context, understanding the qualitative as well as quantitative aspects of tau phosphorylation at more than 80 potential phosphorylation sites is critical for dissecting changes in the landscape of tau phosphorylation during aggregation over the course of disease progression. Mass spectrometry (MS) techniques enable a broad characterization throughout the protein, with high specificity and quantitative precision, which has been challenging using traditional immunochemistry tools.

Over the last two decades, MS sequencing has been primarily used to complement immunoassays for identifying tau phosphorylation sites in tau aggregates (Hasegawa et al., 1992; Hanger et al., 1998). The MS strategies employed have mainly relied on data-dependent acquisitions (DDA), which have provided valuable sequencing data to identify phosphorylated sites (Hanger et al., 2007). However, this proteomic pipeline may lead to stochastically missing tandem MS/MS spectra and thus missing identifications when abundance is low or complex co-eluted phosphorylated peptide patterns cannot be resolved. Moreover, these experiments mainly provided qualitative, dichotomous “detected/undetected” information. Consequently, the abundance of phosphorylation at different confirmed and putative sites and their potential quantitative changes under physiological and pathological conditions remained unclear or undefined.

Furthermore, the limited sensitivity associated with DDA approaches has also prevented the use of MS to quantitatively monitor tau phosphorylation in cerebrospinal fluid (CSF) in which the concentration of tau is three orders of magnitude lower than in the brain (Sato et al., 2018). Tau in the CSF or extracellular tau is released from neurons under certain physiological and pathological conditions (Yamada et al., 2014) and reflects changes in brain intracellular tau (Sato et al., 2018). However, relative tau phosphorylation changes in the CSF are difficult to quantify using immunoassays due to concomitant changes in unphosphorylated tau levels. For example, in AD, tau phosphorylated at T181 (p-tau181) and unphosphorylated, so called “total-tau” (t-tau) are routinely measured by immunoassays; however, the ELISA p-tau181/t-tau ratio does not report the changes in the phosphorylation rate due to the nearly identical information provided by the phospho-tau181 and t-tau ELISA assays (correlations >0.95).

In 2011, the introduction of Quadrupole-Orbitrap hybrid instruments allowed the design of new MS strategies including parallel reaction monitoring (PRM; Gallien et al., 2012, 2014; Peterson et al., 2012). PRM combines the high sensitivity, high-throughput capability, and multiplex assaying of targeted-MS experiments together with the specificity and sequencing ability of high-resolution tandem MS. Applied to tau isoforms measurement, PRM has led to new insights into tau metabolism and truncation (Barthélemy et al., 2016; Sato et al., 2018). We describe how MS using PRM experiments opens new research avenues for understanding the physiology of tau phosphorylation and its alterations in neurodegenerative diseases, particularly in AD.

## Materials and Methods

### Brain Soluble tau Extraction

Brain and CSF studies involving participants were approved by the Washington University Human Studies Committee and the General Clinical Research Center. Written informed consent was obtained from all participants prior to inclusion in the study. Brain soluble tau was extracted as described previously (Sato et al., 2018). Briefly, frozen human brain tissues from controls without amyloid and tau pathologies as described before (Sato et al., 2018) were obtained from Knight Alzheimer’s Disease Research Center (ADRC) at Washington University School of Medicine (St. Louis, MO, USA). Sarkosyl-soluble tau was separated from putative tau aggregates by ultracentrifugation as reported in the literature (Hanger et al., 1998) and pooled. Frozen human brain (200–400 mg, frontal regions) were homogenized in Tris-HCl buffer (25 mM Tris-HCl, 150 mM NaCl, 10 mM EDTA, 10 mM EGTA, 1 mM DTT, phosphatase inhibitor Cocktail 3, Roche Protease Inhibitor, pH 7.4. Final 3.25 mL/mg tissue) on ice. Homogenates were centrifuged at 4°C for 60 min at 11,000× *g*. The supernatant was solubilized in 1% Sarkosyl for 60 min and centrifuged for 2 h at 100,000× *g*. Sarkosyl soluble fractions were pooled and 50 μL fraction was diluted 10 times with 0.5% human plasma before immunoprecipitation. For brain/CSF comparison, brain lysate pool was diluted from 500 to 8,000 times before immunopurification to match CSF tau levels.

### CSF tau Extraction

Human CSF was pooled from a cohort of 80 participants, including amyloid negative and cognitively normal (CDR = 0) controls (*n* = 47, age 60+) and amyloid positive and CDR > 0 AD patients (*n* = 33, age 60+). Five and seven pools of 500 μL CSF aliquots were generated from the control and AD groups, respectively. At the time of initial collection, CSF was spun down at 1,000× *g* for 10 min to remove cell debris and immediately frozen at −80°C. Protease inhibitor cocktail was added during experiments. Tau was immunoprecipitated and desalted as previously described with some modifications (Sato et al., 2018). Briefly, CNBr-activated Sepharose beads (GE Healthcare 17-0430-01) were crosslinked to antibodies Tau1 and HJ8.5, separately at a concentration of 3 mg antibody per gram of beads. Samples are spiked with AQUA peptides (ThermoFisher Scientific) corresponding to 10 fmol phosphorylated and 100 fmol unphosphorylated tau for each sequence of interest per microliter of sample. Tau and p-tau concentration is calculated using these internal standards. Soluble tau was immunoprecipitated in detergent (1% NP-40), chaotropic reagent (5 mM guanidine), and protease inhibitors (Roche Complete Protease Inhibitor Cocktail). Anti-Tau1 and HJ8.5 antibodies conjugated to sepharose beads were diluted 10 and 5-fold, respectively, in inactivated sepharose beads, and 30 μL of 50% slurry of the antibody beads were rotated with the solution for 90 min at room temperature. The beads were washed three times in 25 mM triethyl ammonium bicarbonate buffer (TEABC, Fluka 17902). The bound tau was digested on-beads with 400 ng MS grade trypsin (Promega, V5111) for 16 h at 37°C. Digests were loaded onto TopTip C18 (Glygen, TT2C18.96), desalted, and eluted per manufacturer’s instructions. The eluted peptides were dried by vacuum centrifugation (CentriVap Concentrator Labconco) and were resuspended in 25 μL of a solution of 2% acetonitrile and 0.1% formic acid in MS grade water.

### Mass Spectrometry

A 5 μL aliquot of the peptide resuspension was injected into nano-Acquity LC for MS analysis. The nano-Acquity LC (Waters Corporation, Milford, MA, USA) was fitted with HSS T3 75 μm × 100 μm, 1.8 μm column and a flow rate of 0.5 μL/min of a gradient of solution A and B was used to separate the peptides. Solution A was composed of 0.1% formic acid in MS grade water and solution B was composed of 0.1% formic acid in acetonitrile. Peptides were eluted from the column with a gradient of 2%–20% of solution B in 28 min, then 20%–40% solution B for another 13 min before ramping up to 85% solution B in another 3 min to clean the column. The Orbitrap Fusion Lumos was equipped with a Nanospray Flex electrospray ion source (Thermo Fisher Scientific, San Jose, CA, USA). Peptide ions sprayed from a 10 μm SilicaTip emitter (New Objective, Woburn, MA, USA) into the ion source were targeted and isolated in the quadrupole. These were then fragmented by HCD and ion fragments were detected in the Orbitrap (resolution of 60,000, mass range 150–1,200 m/z). Monitoring of hydrophilic peptides (SSRcalc <9, all without leucine) for peptide profiling was performed on a HSS T3 300 μm × 100 μm, 1.8 mm column at a flow rate of 4 μl/min with an elution occurring with a 2%–12% solution B gradient and a spray operating on a 30 mm SilicaTip emitter.

### Principles of PRM for p-tau Peptides Discovery

**Figure 1:**
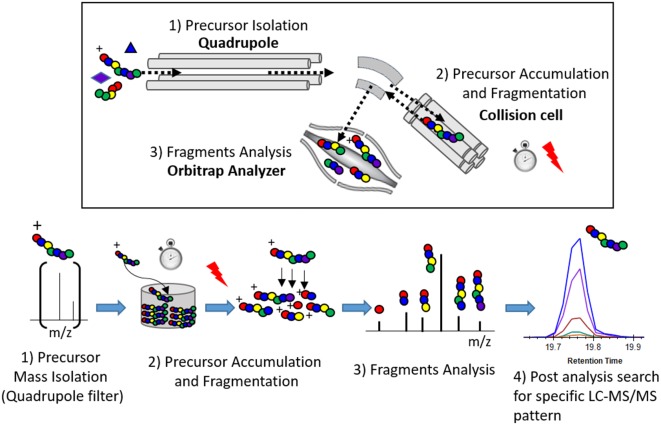
Principle of the parallel reaction monitoring (PRM) experiment.

Tau peptides containing hypothesized phosphorylation sites were generated by digestion of protein from a tau-enriched biological extract such as human brain soluble fraction. These peptides are screened by targeted-MS analysis using a quadrupole-orbitrap instrument, such as the Thermo Fisher Orbitrap Lumos. For each phosphorylated peptide, the selection of targeted-MS parameters such as precursor mass, collision energy, or expected retention time is performed *in silico* and refined using biophysical properties measured for corresponding unmodified peptides that are much more abundant in the sample. For detecting phosphorylated peptides, the quadrupole is set to select a mass window including the hypothesized precursor mass. After precursor collision, all generated fragments are simultaneously measured in the Orbitrap over the time of chromatographic elution. Potentially phosphorylated peptides can be searched in post analysis of the generated data. Skyline software (MacCoss Lab, University of Washington, WA, USA) was used to extract LC-MS/MS data. The hypothetical peptide being screened is detected as a LC-MS/MS fingerprint constituted by the strict co-elution of the extracted masses from predicted MS/MS fragments (Figure 1). The advantage of the PRM method over classic DDA or targeted-MS methods is its ability to use the MS instrument in the highest sensitive configuration possible and conserve discovery capability.

Maximum sensitivity of the Quadrupole-Orbitrap instrument during the screening is essential to detect minor ion fragments and facilitates the identification of phosphorylated peptide together with the localization of phosphorylated sites. Sensitivity of the PRM measurement depends mainly on the number of ions from the target of interest transferred into the Orbitrap analyzer. This number was enhanced by increasing the fill time used to acquire one MS/HRMS scan. The fill time corresponds to the time spent to accumulate the targeted precursor mass from the ion beam into the ion trap device. Accumulated precursors are then fragmented and product fragments are transferred into the Orbitrap to be analyzed. Thus, fill time is set to a maximum limited by the need for a sufficient MS scan rate to acquire enough data points to describe the chromatogram signal (i.e., 8–15 scans per chromatographic peak). Fill time for PRM screening on each investigated phosphorylated peptides was typically set to 1 s.

Fill time is also limited by the risk of trap saturation. This occurs when too many ions are sampled within the same scan and transferred to the Orbitrap, leading to inaccurate mass measurement due to space charge effects. To avoid such saturation, narrow precursor isolation (0.7 Da) together with appropriate sample purification (immuno purification) enriching the target over matrix were chosen to decrease the contribution of potential near-isobaric interferences.

Specificity of the PRM discovery experiment depends on the resolving power of the LC-Q Orbitrap system. Resolving power can be improved by different analytical parameters, with the ultimate goal to obtain interference-free LC-MS/MS fingerprints for the targeted peptides. This limits the risk of ambiguous fragment assignment due to false positive signals. Sample purification preferentially enriching p-tau can also improve the specificity in the discovery of minor p-tau peptides. High chromatographic peak capacity and resolution limit the likeliness of co-elution. A narrow quadrupole isolation window for the precursor decreases the probability of interference on the MS/MS spectrum together with limiting the risk of trap saturation as described above. High Orbitrap resolution and analyzer calibration allow the accurate extraction of mass fragments during data processing limiting, the risk of transitions interference (Gallien et al., 2012; Peterson et al., 2012). The choice of Orbitrap resolution (60 k) is a balance between high resolution requiring more acquisition time and reasonable scan rates compatible with the chromatographic acquisition.

### Quantitative Assessment of Site-Specific Phosphorylation Rate of tau

To quantitatively assess the relative abundance of phosphorylation of specific sites in tau from the brain and CSF, we measured the extent of phosphorylation on each site detected. We used three methods for this purpose: (1) relative comparison between phosphorylated peptide isomers. Signal comparison from transition ions are used to identify each phosphorylated site. This can estimate the relative abundance of each phosphorylated peptides sharing the same sequence; (2) phosphorylated peptides are normalized with the non-phosphorylated peptide as reference. LC-MS/MS transition specific to each phosphorylated peptide is compared to the corresponding transition from the non-phosphorylated peptide. Each phosphorylated site ratio obtained can be compared across the protein sequence. This strategy may be biased by the difference in fragmentation efficiency between the non-phosphorylated and the phosphorylated peptides. This method cannot be applied when the phosphorylated sites are part of a tryptic missed cleavage; and (3) absolute quantitation using internal synthetic labeled standards (i.e., AQUA) for each phosphorylated and non-phosphorylated peptide. Signals from phosphorylated and non-phosphorylated standards are used to define an internal ratio. This strategy takes into account the fragmentation specificity of each compared peptide but requires peptides synthesis for each monitored species.

In this study, except for those including missed trypsin cleavage, the second method was utilized to calculate phosphorylation rates. We also applied the third method (AQUA normalization) on a limited set of phosphorylated sites found in both brain and CSF extracts when synthetic phosphorylated peptides were available (i.e., T175, T181, S199, S202, T205, T217 and T231) and one site found only in brain (S404; Table 1). For AQUA measurement, brain extracts were diluted 500–8,000 times to be comparable to CSF tau levels. This dilution minimized the matrix effect that may result from significantly different ratio of AQUA internal standard to tau peptide levels in the brain vs. CSF. The first method was used for initial interpretation of the complex LC-MS/MS patterns from p-tau sequence containing numerous phosphorylated residues.

**Table 1:** Brain/cerebrospinal fluid (CSF) pool phosphorylation rate comparison (HJ8.5+Tau1 IP-MS).

Phosphorylated site	Peptide sequence	Isoform	Brain lysate soluble (1 ml 10×)	Brain lysate soluble (1 ml 500×)	Normal CSF (500 μl)	AD CSF (500 μl)
S46	45–67	1N/2N	?	nd	x	x
T50	45–67	1N/2N	0.5	nd	x	x
T52	45–67	1N/2N	2.2	nd	x	x
S56	45–67	1N/2N	0.01	nd	x	x
S61/T63/S64 (2 sites minimum)	45–67	1N/2N	0.5/0.2	nd	x	x
S68	68–126	1N	1.4/0.3	nd	x	x
	68–87	2N	0.3/0.05	nd	x	x
T69	68–126	1N	1.4/0.3	nd	x	x
	68–87	2N	0.3/0.05	nd	x	x
T71	68–126	1N	0.2	nd	x	x
	68–87	2N	0.02	nd	x	x
T76	68–87	2N	0.01	nd	x	x
T95	88–126	2N	?	nd	x	x
T101	88–126	2N	0.06–0.22	nd	x	x
T102	88–126	2N	0.06–0.22	nd	x	x
T111	103–126	0N	0.02	nd	0.8	8.1
	68–126	1N	0.2	nd	x	x
	88–126	2N	0.07	nd	x	x
S113	103–126	0N	0.2	nd	x	low
	68–126	1N	0.53	nd	x	x
	88–126	2N	0.59	nd	x	x
T123	103–126	0N	0.02	nd	x	x
	68–126	1N	0.02	nd	x	x
	88–126	2N	x	nd	x	x
T153	151–155	all	x	nd	x	0.8
T175 mc	171–180	all	nd	0.1*	x	0.1
T181 mc	175–190	all	nd	9.5*	10.1*	13.3*
S184/S185	181–190	all	0.1	nd	x	x
S199 (1)	195–209	all	0.29	1.3*	0.2*	0.2*
S202	195–209	all	3.9	9.7*	2*	1.5*
S199+S202	195–209	all	0.02	nd	x	x
S198+S202	195–209	all	0.01	nd	x	x
T205	195–209	all	x	x	2.3*	4*
S208	195–209	all	x	x	0.001	0.003
T212	210–221	all	?	nd	x	
S214	212–221	all	0.14	0.08	0.04	0.07
T217	212–221	all	0.43	0.5*	1.9*	8.5*
T231 mc	226–234	all	nd	0.1*	0.8*	1.4*
S396	396–406	all	1.2	nd	x	x
S404	396–406	all	95	110*	x	x
S396+S404 mc	386–406	all	nd	nd	x	x
S409	407–438	all	0.3	nd	x	x
S411/S412/T413 (2 sites minimum)	407–438	all	1.2	nd	x	x
S416	407–438	all	1.0	nd	x	x
Number of unique sites			29		9	12

Values indicate phosphorylated/phosphorylated ratio in %. x, not detected. *measured using AQUA internal standards. nd, not determined. ?, not confidently assigned. (1) likely underestimated due to lower recovery using Tau1 immunocapture.

### Statistics

Data are represented as mean ± SD unless otherwise specified. After confirming the normal distribution of the data, one-way ANOVA followed by *post hoc* analyses (Tukey test) were performed for comparing tau phosphorylation rates. Additional statistical analysis was completed using GraphPad version 8.0.1 (244) from GraphPad Software Inc. Statistical significance between relevant groups was determined with a two-tailed, unpaired, Mann-Whitney *t*-test. Significance was evaluated at the 0.01 and 0.05 level.

## Results

### Phosphorylation Sites on tau Protein in the Normal Non-AD Human Brain

In order to determine phosphorylation sites of normal soluble brain tau, extracts from healthy controls were purified by immuno capture using Tau-1 and HJ8.5 tau antibodies concentrating both phosphorylated and unphosphorylated tau. The tryptic digestion of brain tau isoforms generated 27 unmodified peptides that were long and hydrophobic enough to be detected by LC-MS (Barthélemy et al., 2016). Twenty-five peptides contained serine and/or threonine as potential phosphorylation sites. Several peptides contained multiple potential phosphorylation sites (4–9) leading us to consider different mono-phosphorylated peptides potentially co-eluting together during the LC separation. After this, the PRM screening method described above was applied to these peptides.

**Figure 2:**
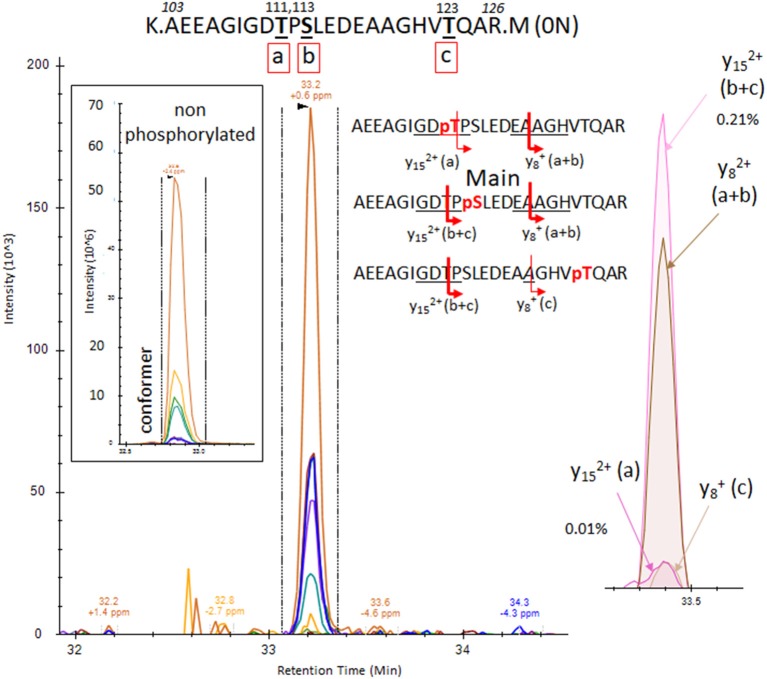
PRM screening of the mono-phosphorylated tau (p-tau) sequence at 103–126 (0N isoform). We identified a unique LC-mass spectrometry (MS)/MS pattern eluting closely to the unmodified peptide 103–126 and containing fragment series expected for phosphorylation at T111 (a), S113 (b) or T123 (c). Hypothetical y ion fragments from each p-tau peptide are underlined on the sequences. We deconvoluted the potential co-elution of the three putative mono-phosphorylated peptides. Ion fragment y15 without phosphate is specific to the phosphorylated peptide on residue T111 [y15 (a)] and the y8 fragment with phosphate is specific to p-tau peptide on residue T123 [y8 (c)]. Corresponding extracted ion chromatograms (XIC) are detected in low abundance above the limit of detection, supporting the identification of the two corresponding mono-p-tau peptides. In contrast, the y15 fragment with phosphate, shared by pT111 and pS113 [y15 (a+b)], and the y8 fragment without phosphate, shared by pS113 and pT123 [y8 (b+c)], are much more abundant. These signal differences support the existence of the tau peptide mono-phosphorylated at residue S113 (b) as the main component of the pattern.

**Figure 3:**
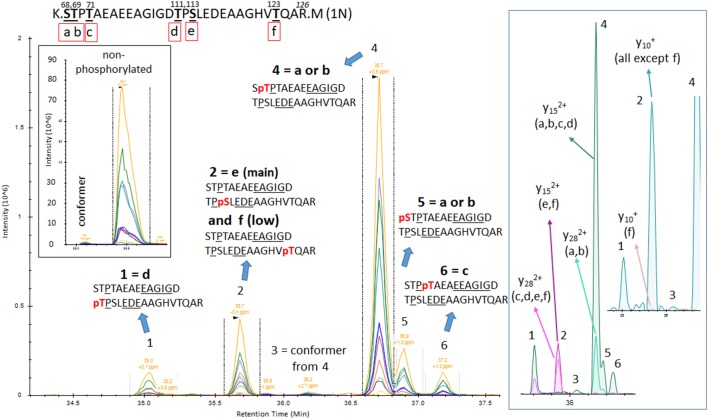
PRM screening of mono-p-tau sequence 68–126 (1N isoform) containing six potential phosphorylation sites. Three phosphorylation sites are shared by peptides containing residues 103–126 as described in Figure 2 (d–f). Six LC-MS patterns were identified. The Y28 fragment carrying phosphate, shared by pS68 (a) or pT69 (b), is found in the two LC-MS patterns 4 and 5. This demonstrates the existence of the two phosphorylated peptides but corresponding LC-MS patterns cannot be strictly assigned without the detection of ion fragment y29 to differentiate pS68 and pT69. Specific fragments corresponding to pT71 (c) and pT111 (d) are found in LC-MS patterns 6 and 1, respectively. Specific fragments for pS113 (e) and pT123 (f; y15 with phosphate) are found in the LC-MS pattern 2. This pattern contained both y10 fragments with and without phosphate, suggesting the co-elution of these two phosphorylated peptides. Since y10 without phosphate has the major signal in comparison to y10 with phosphate, the degree of pT123 is likely lower than pS113. LC-MS pattern three is attributed to a minor conformer or LC artifact from the phosphorylated peptide from pattern four as found for the non-phosphorylated peptide.

**Figure 4:**
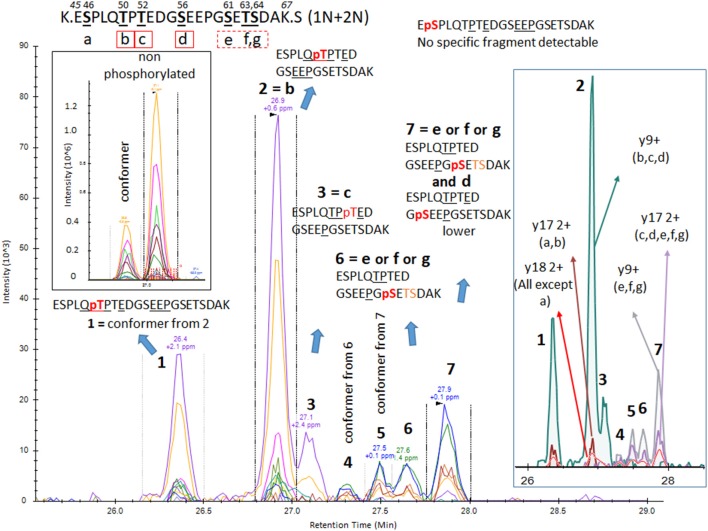
PRM screening of mono-p-tau sequence 45–67 (1N and 2N isoforms). A strong signal from a conformer was identified on the front of a non-phosphorylated peptide LC-MS pattern. Thus, we predicted that corresponding LC-MS patterns for phosphorylated peptides would also have conformers separable by LC. Indeed, PRM scan interpretation led to the detection of pT50 (b) as the main phosphorylation site on this sequence (pattern 2). Pattern 1, with a similar fragmentation fingerprint, was attributed to a conformer of pT50. pS46 (a), able to differentiate signals from pT50 (b), was not detected, suggesting this phosphorylation would be absent or low and co-eluted with other phosphorylated peptides sharing similar non-specific fragments. Co-elution of y9, y15 without phosphate, and y17 with phosphate in LC-MS pattern 3 identified a phosphorylation on residue T52 (c). Patterns 4/6 and 5/7 were respectively paired as conformers. Thus, two phosphorylated peptides could not be separated. Fragments found in these patterns were consistent with phosphorylation on one of the S61 (e), T63 (f), and S64 (g) residues. MS/MS intensities were insufficient to identify the sites but at least two of these three sites were likely phosphorylated on this sequence. Additionally, a minor y9 fragment without phosphate was found in the shoulder of pattern 7, which could be attributed to minor phosphorylation on residue S56 (d).

**Figure 5:**
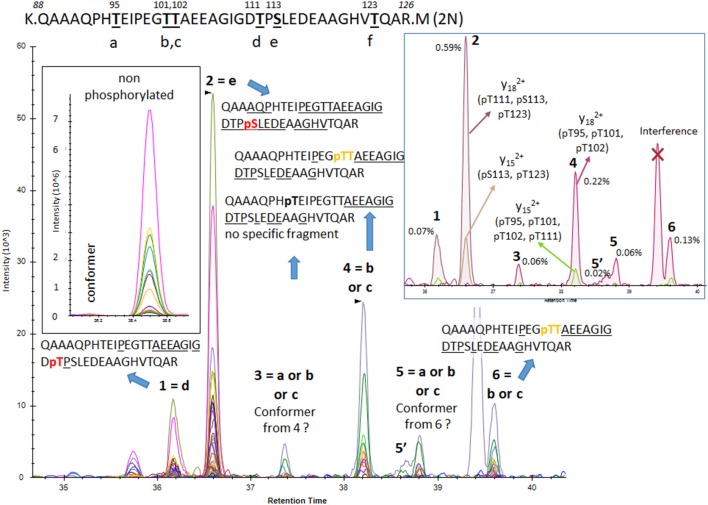
PRM screening of mono-p-tau sequence 88–126 (2N isoform). Six potential phosphorylation sites are located in this sequence and six LC-MS patterns were identified. Fragments found in pattern 1 and 2 were consistent with phosphorylated peptides at residues T111 (d) and S113 (e), respectively. No specific fragment from the phosphorylated peptide at residue T123 (f) was found. Patterns 4 and 6 contained a low signal of the y29 fragment matching with phosphorylation on residue T101 (b) or T102 (c), but no specific fragment able to differentiate them was detected. Patterns 3 and 5 shared fragments found in patterns 4 and 6 but in lower abundance, locating the phosphorylated residue at the N-terminus on residue G109. This could indicate the presence of an additional phosphorylated peptide, likely at residue T95 (a) or abundant conformers from peptides found in patterns 4 and 6.

**Figure 6:**
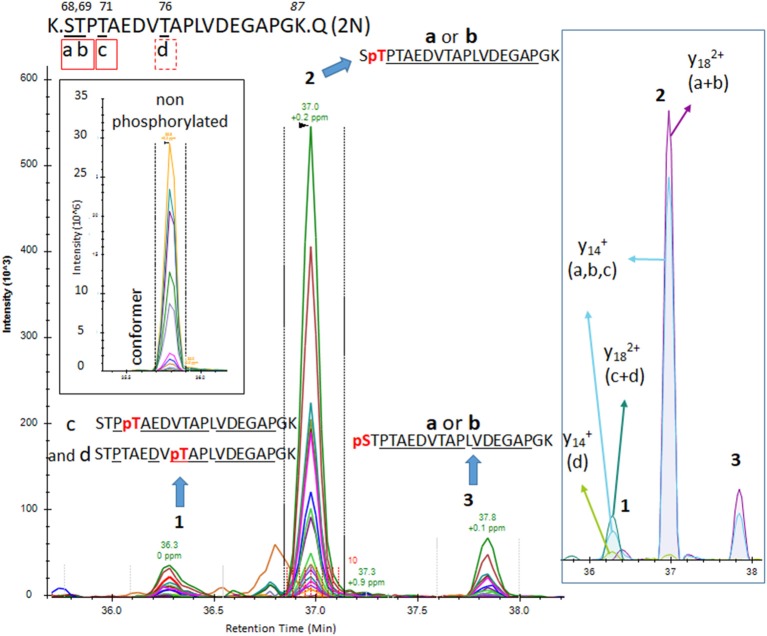
The PRM scan of mono-p-tau sequence 68–87, containing four potential phosphorylation sites. We detected three LC-MS patterns. Patterns 2 and 3 were consistent with phosphorylation at residues S68 (a) or T69 (b). Pattern 1 contained both fragments compatible with the presence of two co-eluted phosphorylated peptides at T71 (c) and T76 (d). Comparison of y14 XIC with and without phosphate in pattern 1 indicates pT71 (c) is more abundant than pT76 (d).

The results from analysis of the projection domains in the tau sequence (103–126) revealed multiple p-tau peptides eluting into overlapping complex LC-MS/MS patterns (Figures 2–6). We identified three phosphorylation sites from the 0N isoform, the shortest isoform of the tau protein. Although the LC system did not have the resolution for differentiating the three phosphorylated peptides, fragment identification and corresponding intensities were used to deconvolute a signal composed of one major phosphorylation site at residue S113, and two minor phosphorylation sites at residues T111 and T123 (Figure 2). In contrast, when screening tau sequences from the longer 1N and 2N isoforms, further chromatographic separation was necessary to simplify phosphorylated peptide elution patterns, especially when multiple potential mono-phosphorylation sites were predicted within the same peptide sequence (Figures 3–5). LC separation allowed us to identify co-eluted, semi-specific MS/MS fragments which contained differentially phosphorylated residues. However, we have also identified a few signals that could result from LC artifacts, likely due to dual conformations of the same peptide in solution (Figure 4). This effect was important for peptide sequence containing amino acid residues 45–67 (in the 0N isoform) for both unmodified and phosphorylated peptides (Figure 4) and less prevalent for sequences 68–126 (1N) and 88–126 (2N; Figures 3, 5).

**Figure 7:**
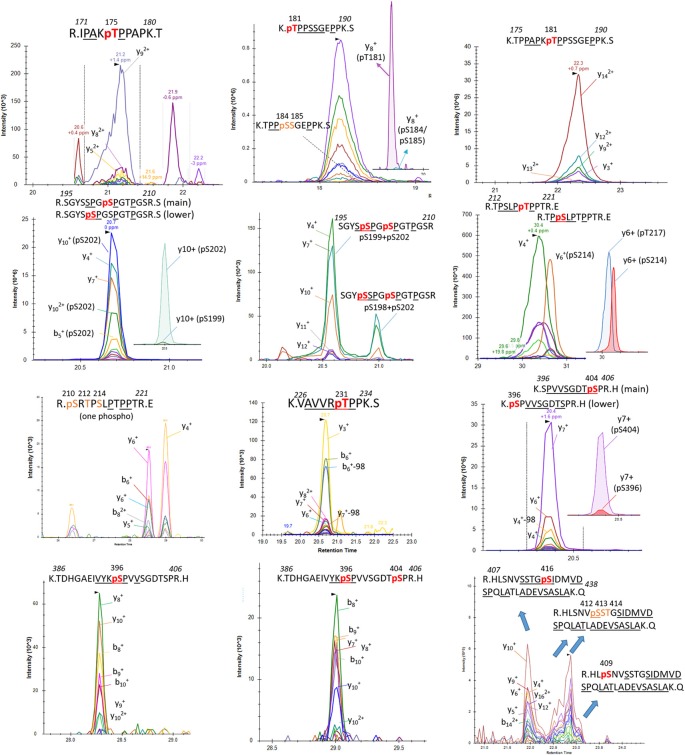
Detection of phosphorylation sites in the mid-domain and C-terminus of brain p-tau protein.

Phosphorylations at residues S113, T111 and T123 were confirmed on peptide sequences 68–126 (1N) and 88–126 (2N). In all cases, the S113 signal was the most abundant of the three with a phosphorylation rate of 0.2%–0.5% (Figures 3, 5, Table 1). Phosphorylations at residues T50, T52 and S56 were clearly identified on the peptide sequence 45–67 (shared by 1N and 2N isoforms). On the same peptide, LC-MS signals indicated at least two of the three residues (S61, T63, and S64) were phosphorylated (Figure 4). No specific signals were found to identify potential phosphorylation at residue S46. Phosphorylation at residues S68 and T69 were evidenced in sequences 68–126 (1N) and 68–87 (2N) but no specific fragments were detected to differentiate their LC-MS patterns. On the same 1N and 2N peptide sequences, lower phosphorylation levels were detected on T71. 2N specific phosphorylations on the T76, T101, and T102 residues were also identified (summarized in Figure 7).

Induction of trypsin missed cleavage was also considered for the screening of phosphorylated residues located after a tryptic site as reported previously (sites T175, T181, S212, T231 and S396; Hanger et al., 1998). Our PRM screening successfully detected LC-MS patterns corresponding to p-tau peptides already described in normal brain tissue by Hanger et al. (1998; T181, S199, S202 and S404; Figure 7). Corresponding LC-MS signals were high, suggesting that p-tau peptides previously reported in normal human brains are likely the most abundant. Our comparison of S199 and S202 phosphorylation indicated a much more prevalent abundance of phosphorylation at S202 (Figure 7). The use of an anti-Tau1 antibody for tau extraction, associating with a non-phosphorylated epitope on the 192–199 amino acid sequence, could explain the low recovery of S199 phosphorylation in the extract. Given the abundance of S202 and S404 phosphorylation in the extract, we searched for the presence of di-phosphorylated peptides from sequences 195–209 and 386–406. We detected two LC-MS patterns with specific fragments corresponding to double phosphorylation at S202/S199 and S202/S198 and one LC-MS pattern corresponding to a double phosphorylation at 396/404 (Figure 7).

Additional screenings of soluble tau in brain extract evidenced other less abundant mono-phosphorylated peptides corresponding to phosphorylated residues at T175, S214, T217, T231, and S396. Signals from fragments corresponding to a phosphorylated peptide at S184 or S185 were also detected at low levels when the mono-phosphorylated peptide sequence 181–190 was screened (Figure 7). The search for mono-phosphorylation on the long sequence 407–438 discovered a pattern consistent with phosphorylation at residues S409 and S416 and at least one phosphorylation on the group of residues S412/S413/T414.

Overall, we identified a minimum of 29 unique phosphorylation sites detectable in the soluble tau fraction extracted from normal non-AD brains using the PRM screening method (Table 1). Twenty-five of them can be unambiguously assigned to unique LC-MS signals and four additional phosphorylation sites were evidenced without assignment to the exact LC-MS patterns. These sites were located on three clusters: a minimum of 14 phosphorylated sites were located in the N-terminal projection domain, 10 on the proline-rich domain in the middle of the sequence, and six on the C-terminus.

### Phosphorylation Sites on tau Protein in the CSF

In comparison to the brain tau digest, CSF tau purification using tau-specific antibodies and digestion generated detectable peptides mainly from the mid-domain of the protein sequence (residues 150–221). Peptides were detectable to a lesser extent from the N-terminus, and almost no sequence was detectable from the microtubule binding repeat (MTBR) domain or the C-terminus of tau (Barthélemy et al., 2016; Sato et al., 2018). This difference in signal recovery may result from tau truncation during its release from neurons (Sato et al., 2018). The peptide recovery of this mid-domain was sufficient to monitor corresponding minor phosphorylated isoforms using the current PRM method. Conversely, a significant technological advance in MS method will be required to detect phosphorylated peptides in the MTBR and C-terminal domains.

**Figure 8:**
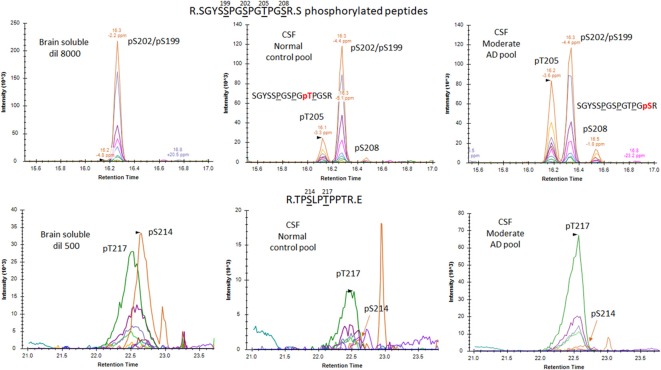
Phosphorylated peptide profiles from tau sequences 195–209 and 212–221 are variable between the soluble brain fraction, normal cerebrospinal fluid (CSF), and Alzheimer’s disease (AD) CSF tau protein. Brain soluble tau extracts are diluted as indicated to approximately match corresponding CSF tau level. Phosphorylated peptides on 195–209: in brain lysate, one signal corresponding to the co-elution of two phosphorylated peptides pS199 and pS202 is observed. In CSF, two additional signals are observed. Fragment analysis allowed the assignment of the signal on left to pT205. In AD CSF, the two signals are increased allowing the identification of specific fragments assigning the signal on the right to pS208. Phosphorylated peptides on 212–221: two signals with similar MS intensities corresponding to pT217 and pS214 are identified in brain lysate. In CSF, the signal corresponding to pT217 is the most intense while pS214 is close to the limit of detection, indicating a dramatic change in their relative abundance in comparison to the brain extract. In AD CSF, pT217 is significantly increased due to specific hyperphosphorylation.

PRM screening of tau phosphopeptides from normal control CSF identified several phosphorylation sites in common with brain soluble tau at T181 (not shown), S199, S202 and T217 (Figure 8). A low signal corresponding to pS214 was also detected (Figure 8). A specific fragmentation pattern corresponding to pT205 was identified in the CSF extract and was separated by chromatography from the pS199/pS202 signals (Figure 8).

**Figure 9:**
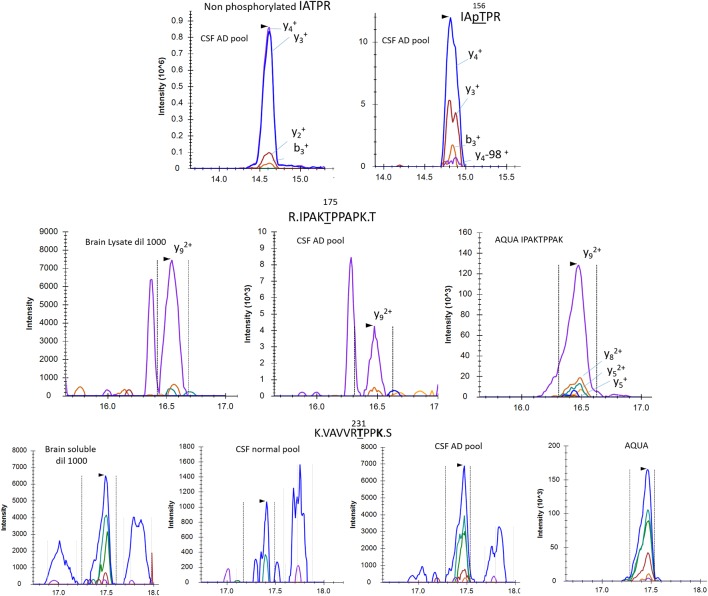
pT153, pT175 and pT231 phosphorylated peptides identified in CSF. AQUA internal standard signals are shown for pT175 and pT231. Fragmentation pattern of pT153 is similar to unmodified.

**Figure 10:**
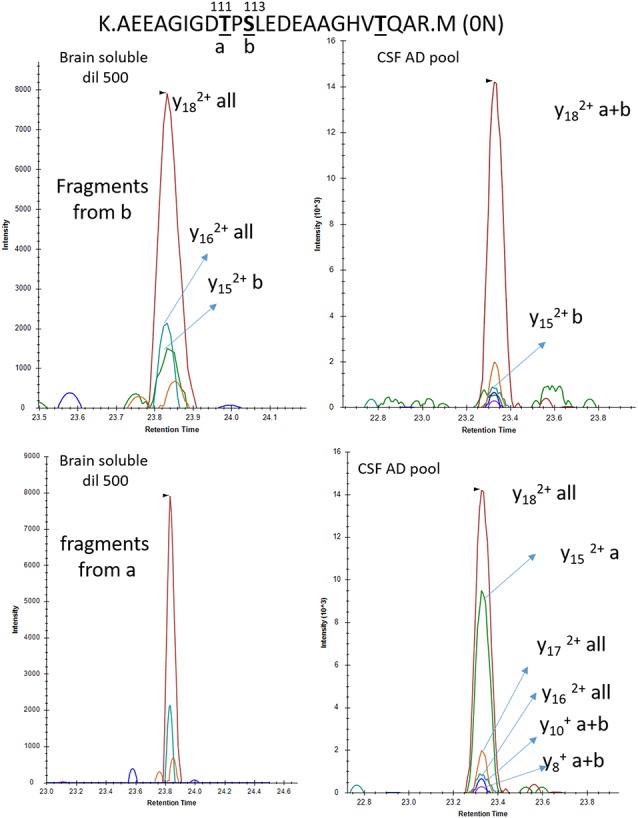
Phosphorylation abundance on T111 is higher in the CSF than the brain relative to S113 phosphorylation. In both brain and CSF, the MS/MS fragment y18 common to all mono-phosphorylated peptides on tau sequence 103–126 is detected. The relative abundance of the y15 fragment from pS113 (b) is significantly lower in CSF in comparison to brain extract. Inversely the y15 fragment from pT111 (a) is abundant in CSF and not detectable in brain soluble extract diluted to match the AD CSF tau level.

**Figure 11:**
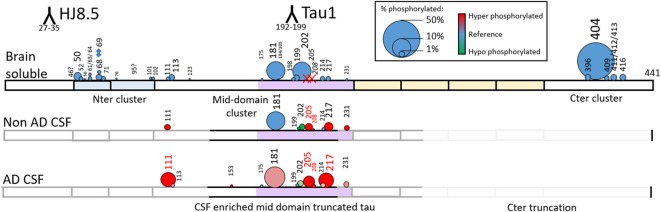
Relative abundance of tau phosphorylation depends on the biological extract and varies across the protein sequence. Comparison of tau phosphorylation abundance measured by MS in normal brain lysate, normal CSF and AD CSF extracted by immunocapture using HJ8.5 and Tau1. Circle area is proportional to site phosphorylation abundance. Red and green colors indicate an increase or decrease, respectively, in comparison to the brain soluble profile taken as reference (blue). Tau is c-terminally truncated in CSF, which explains the absence of detection of the C-terminal cluster of phosphorylation sites. Phosphorylation on T205 and S208 is specific to CSF (red X on Brain Soluble—top).

To increase the probability of detecting additional phosphorylated sites in CSF tau, we analyzed CSF pools from AD patients with mild to moderate dementia. We expected AD CSF pools would contain increased concentrations of tau as well as increased levels of p-tau. The same phosphorylated residues found in normal CSF (T181, S199, S202, T217 and T231) were detected in AD CSF. Additionally, signals corresponding to pS113 and pT175 previously found in tau from the brain but not in the normal CSF were detected in AD CSF (Figures 8, 9). A LC-MS/MS pattern containing specific fragments and distinct retention times from S202/S199 phosphorylated peptides allowed the identification of a new phosphorylated peptide at S208. When we reexamined pS208 in normal CSF, we detected the corresponding signal in low abundance. A LC-MS/MS pattern matching with a new phosphorylated peptide at residue T153 was detected close to the level of corresponding unphosphorylated peptide (Figure 9). Careful reexamination of brain extract data suggested the presence of a low abundant signal corresponding to this site. Finally, we found specific signals corresponding to phosphorylation on the 103–126 amino acid sequence from the 0N isoform in AD CSF, indicating S111 as the main phosphorylation site on this peptide, while S113 was barely phosphorylated (Figure 10). Altogether, 12 phosphorylation sites were detected in CSF tau, two of which were not detectable in brain lysate (Figure 11).

### P-tau Abundance in Brain

In the brain, S404 was the most highly phosphorylated of the examined phosphorylation sites (pS404/S404 = 110%, i.e., 52% of S404 is phosphorylated). Thus, more than half of the brain tau was phosphorylated at S404 compared to other highly phosphorylated sites at S202 and T181 (9.7% and 9.5%, respectively). 1.3% of S199 was phosphorylated but this could be underestimated due to the inability of the Tau1 antibody used for extraction to bind its corresponding phosphorylated epitope (Liu et al., 1993). Phosphorylation on C-terminal sites other than S404 was found to be around 1%. On the N-terminus, T52, S68/T69, T50, and S113 were also phosphorylated with abundances ranging from around 0.5% to 2%. Other detected sites appeared to be phosphorylated at much lower levels (<0.5%).

### CSF p-tau Abundance Measurements Highlight Differences Compared to the Brain

**Figure 12:**
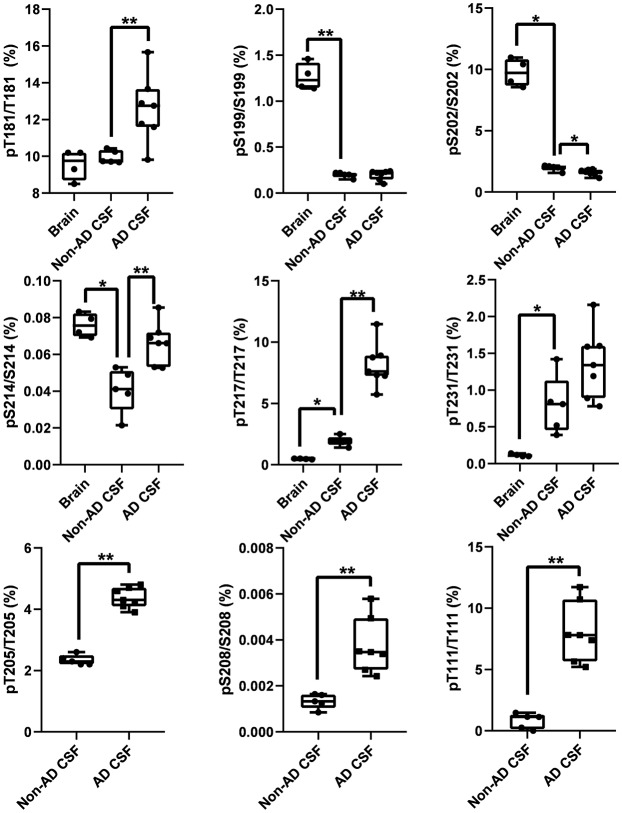
Tau phosphorylation sites are differentially modified in brain, normal CSF and AD CSF. Measurements are the relative abundances of the phosphorylated signal compared to the corresponding nonphosphorylated site (HJ8.5+Tau1 IP-MS). Brain results are obtained from diluted lysates from 500× to 8,000× factors to match the CSF tau level. Phosphorylations on T205 and S208 are undetectable in brain tissue. Phosphorylation on T111 is undetectable in 500x diluted lysate but was detected in 10× diluted lysate with a corresponding abundance of 0.02%. Legend: **indicates significance at *p* = 0.01 level and *indicates significance at *p* = 0.05 level.

In addition to the unique detection of pT205 and pS208 in CSF tau but not brain, the relative phosphorylation abundance of certain other phosphorylation sites detected in brain tau was altered in comparison to control CSF tau (Figure 12). Compared to brain, CSF tau phosphorylation was significantly lower at sites pS199 (6-fold decrease, *p* = 0.008), pS202 (5-fold decrease, *p* = 0.016), and pS214 (2-fold- decrease, *p* = 0.016). Conversely, CSF tau phosphorylation was significantly higher at sites pT217 (4-fold increase, *p* = 0.016), pT231 (7-fold increase, *p* = 0.016) and pT111 from 0N (16-fold increase). PT181 abundance was similar in the CSF and the brain (~10%). When measured in AD CSF, pT175 remained low (0.1%–0.2% compared to 0.1% in brain).

**Figure 13:**
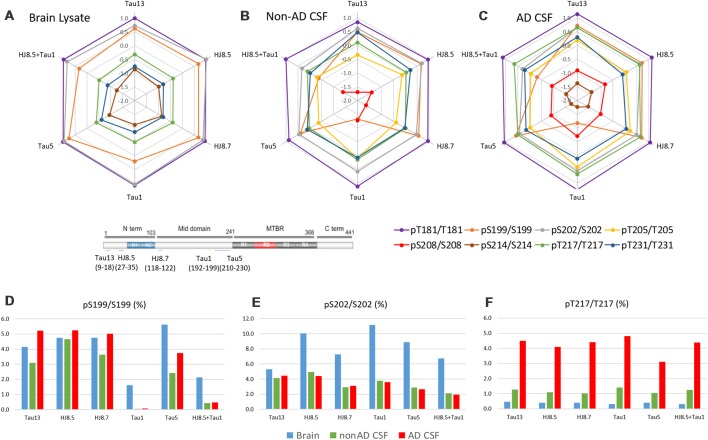
Antibody effect on phosphorylation ratio measurement by IP-MS. Brain lysate pool, non-AD (*n* = 1) and AD CSF (*n* = 1) pools were immunoprecipitated in parallel with antibodies against tau N-terminus projection domain (Tau13 or HJ8.5) or mid-domain (HJ8.7, Tau1 or Tau5). Radar plots of phosphorylation ratios measured on main CSF sites (log_10_ scale) are shown in brain lysate **(A)**, non-AD CSF **(B)** and AD CSF **(C)**. **(A–C)** Tau phosphorylation ratio measurements on pT181/T181, pT231/T231 are consistent across antibodies tested. PS199/S199 is decreased by using Tau1 or Tau1+HJ8.5 in comparison to other antibodies. **(D)** Low recovery of pS199 by Tau1 or Tau1+HJ8.5 IP underestimate pS199/S199 ratio measurements compared to other antibodies tested. Tau13, HJ8.5, and HJ8.7 antibodies indicate no significant changes of pS199 phosphorylation ratio between brain and CSF. Compared to brain, pS202/S202 CSF hypophosphorylation **(E)** and pT217/pT217 hyperphosphorylation **(F)** are evidenced independently of the antibody used for IP-MS.

Brain and CSF tau have different truncation patterns: brain tau isoforms are mainly full length while CSF tau isoforms are truncated. Brain and CSF tau isoforms and corresponding peptides recovered after IP depend on the antibody used for the immunoprecipitation (Sato et al., 2018). Similarly, antibodies used to immunoprecipitate tau could impact tau phosphorylation recovery. To evaluate this impact on p-tau recovery, we compared IP-MS results on phosphorylation rates using different antibodies (Tau13, HJ8.5, HJ8.7, Tau1 and Tau5) in addition to the Tau1+HJ8.5 combination (Figure 13). A significant decrease of pS199/S199 ratio was observed when Tau1 or Tau1+HJ8.5 were used in brain and CSF. This suggests CSF truncation between the N-terminus and the mid-domain could affect pS199 phosphorylation measurement compared to brain. PS199/S199 rate measured in brain and CSF with other antibodies appeared to be relatively similar. Interestingly, some reactivity was still observed for pS199 in brain extract and to a lesser extent in CSF, suggesting nonspecific binding of Tau1, though S199 phosphorylation is present at the end of the reported Tau1 epitope (192–199). The other results were confirmed independent of the antibody used for immunoprecipitation.

Since phosphatase activity during the post-mortem interval (PMI) before autopsy can decrease tau phosphorylation measured in brain extracts, we investigated the impact of the PMI on phosphorylation rates commonly found in CSF and brain extracts. Ten brains samples (middle frontal gyrus) without tau pathology collected from participants with PMI ranging from 5 to 16 h were analyzed. None of the brain extracts originally analyzed had a PMI of greater than 16 h. We did not find significant association (Spearman test, 95% confidence interval, not shown) between PMI and phosphorylation rates measured on T181, S199, S202, S214, T217, T231 and S404 sites.

### AD Specific p-tau Change in CSF

**Figure 14:**
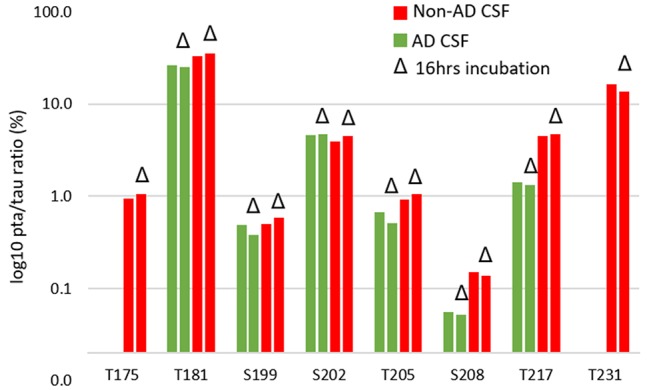
CSF incubation does not impact phosphorylation rate measurement on tau.

The increased CSF p-tau commonly reported in AD could be the consequence of two potential effects: (1) the global increase of tau regardless of its phosphorylation status; and (2) increased hyperphosphorylation at specific sites. Normalizing p-tau signal or level using non-phosphorylated tau allows for quantifying changes in phosphorylation stoichiometry (i.e., hyper- or hypo-phosphorylation) occurring at specific sites independently from the global change in t-tau. The use of “hyper- and hypo- phosphorylation” refers to a change in the ptau/tau ratio in comparison to a ratio measured for a selected reference. For example, we describe hyperphosphorylation of normal CSF in comparison to brain measurements or hyperphosphorylation of AD CSF in comparison to normal CSF. As we recently demonstrated, soluble tau production is increased in AD and correlates with amyloid plaques. Therefore, controlling for not just the *amount*, but the *rate* of phosphorylation is key to understand. pT181, pT217, pT231, pT205, pS208 and pS214 were hyperphosphorylated in AD CSF as was 0N-specific pT111 (Figure 12). PT153 and pT175 were not detected in non-AD and likely increased slightly in AD. The sites showing significant hyperphosphorylation compared to non-AD were pT181 (*p* = 0.010), pS214 (*p* = 0.005), and pT217 (*p* = 0.003). pT181, pS214, and pT217 showed approximately 1.2-fold, 1.6-fold, and 4-fold increases in phosphorylation respectively. Interestingly, not all the monitored sites were hyperphosphorylated: pS199/S199 was not significantly changed and pS202/S202 was significantly lower (*p* = 0.030) with an approximately 1.2-fold decrease. Robustness of phosphorylation ratios was assessed by incubating CSF for 16 h. Incubation did not significantly affect tau phosphorylation ratio differences observed between non-AD and AD CSF (Figure 14). Moreover, absence of kinase activity on CSF tau was confirmed by the non-detectability of phosphorylation on recombinant 15N-tau spiked in CSF after incubation (not shown).

## Discussion

### Technological Advancement of PRM on p-tau Measurement

We report the most comprehensive qualitative and quantitative analyses of p-tau in normal brain tissue and CSF to date. Our approach contrasts with previous DDA studies on tau phosphorylation which may not have captured potential minor phosphorylation sites. However, our approach uses highly sensitive targeted-MS in PRM mode to detect and quantify minor phosphorylation. Phosphorylation site identification depends on the careful manual interpretation of LC-MS/MS patterns to identify co-elution of specific ion fragments for each examined phosphopeptide. In prior studies, brain tau phosphorylation has been mainly investigated in insoluble extracts enriched in hyperphosphorylated tau from PHF and, to date, the most detailed study has reported nine phosphorylation sites in normal human tau protein (Hanger et al., 2007). Our in-depth PRM data analysis detected more than 29 phosphorylated residues with a majority being very low in abundance. Indeed, the previously reported nine sites were amongst the most abundant modifications in the protein. Application of PRM analysis to normal CSF tau, present in much lower abundance compared to the brain, led to the detection of nine phosphorylation sites initially, with three additional sites detected in AD CSF.

In addition to the significantly increased number of phosphorylation sites detected, we demonstrated how sensitive PRM screening enabled us to quantitatively assess the tau phosphorylation rate or stoichiometry, independent of global tau concentration. This concept is frequently overlooked but critical in assessing changes in AD when absolute CSF p-tau concentration may increase solely due to increase in global tau isoforms concentration, and not due to change of relative p-tau abundance. Phosphorylation rate measurement from this study enabled for the first time the comparison of the degree and distribution of phosphorylation rate changes (hyper- vs. hypo-phosphorylation) across the protein, in different compartments (intracellular brain vs. extracellular CSF), and in different pathological conditions (AD vs. non-AD).

Some caveats of the PRM screening process are the low throughput for discovery and the risk of missing p-tau species or other post-translational modification (PTM) not hypothesized in the study. Identifications from MS data can be further used in a larger validation or clinical cohort to design scheduled LC-MS methods, increasing multiplexing and throughput (Gillette and Carr, 2013). Other proteases such as AspN could provide a different set of tau and p-tau peptides than from trypsin digest, allowing for a better coverage of tau, i.e., the C-terminal domain (Hanger et al., 1998; Sato et al., 2018). The search could be further refined by screening additional doubly- or triply-phosphorylated tau peptides not considered in this study, although their abundance may be minimal unless there is biological coordination of site phosphorylation.

### Identification of a Cluster of Phosphorylation Sites on the tau Projection Domain

We found a cluster of previously undescribed phosphorylated residues on the N-terminus projection domain of tau containing alternative splicing-dependent peptides. Interestingly, this domain was not previously found to be extensively phosphorylated in PHF in the insoluble human brain fraction (Hanger et al., 2007; Thomas et al., 2012; Funk et al., 2014; Russell et al., 2016). This cluster was also not extensively characterized in the recent comprehensive study performed in mouse tau protein in the murine brain (Morris et al., 2015). These discrepancies could be attributed to the difficulty of characterizing complex mixtures of phosphorylated peptides, distinguishable only by few specific MS/MS fragments and/or subtle retention time shifting on LC. For example, S46 is the only phosphorylated site previously reported in this domain in normal human tau (Hanger et al., 1998). However, we did not detect a specific signal for this species, which can be confounded by search algorithms with neighboring phosphorylated sites at T50 or T52, sharing a close fragmentation pattern. In this regard, manual inspection is essential to clearly interpret and decipher corresponding LC-MS/MS patterns for each phosphorylated site. Another possible explanation of this discrepancy could be the relatively low abundance of the N-terminal domain in PHF in comparison to the MTBR domain, the mid-domain, and the C-terminus (Mair et al., 2016).

This N-terminus projection domain has been recently assigned as a part of the dominant component of the repulsive barrier that prevents neighboring microtubules (associated to tau *via* the MTBR domain) from getting close to each other (Chung et al., 2016). This sequence contains numerous acidic residues and an increase in phosphorylation may contribute to increased global acidity, enforcing the repulsive barrier. Together with a variable amount of N-terminal extension induced by alternative splicing on exon 2–3, tau phosphorylation could regulate tau/tau N-terminal interactions and microtubule intermolecular distance.

### Biological Implications of Different p-tau Profiles in the Brain vs. CSF

Tau is mainly an intracellular protein that functions in microtubule stability and was traditionally considered to only be released extracellularly upon nerve injury or cell death. However, recent studies have suggested that tau is secreted under physiological and pathological conditions in a regulated manner (Karch et al., 2012; Yamada et al., 2014). By comparing soluble brain tau and CSF tau profiles in parallel to intracellular and extracellular tau profiles and metabolism in neuronal models, we have recently shown that tau secretion is an active process that involves different turnover rates of tau isoforms including truncated tau and p-tau (Sato et al., 2018). Understanding the association between this active secretion and phosphorylation of tau through comparison between brain and CSF p-tau profiles provides potential insight into AD pathogenesis.

We speculate that p-tau isoforms enriched in the CSF have less affinity for microtubules and a higher likelihood to be secreted. Inversely, p-tau isoforms impoverished in the CSF could have more propensity to stay inside the neurons and/or avoid cleavage. Our results demonstrate that several phosphorylated residues are significantly enriched in the CSF compared to brain extracts, such as T217, T231, T153 and T111. All are proline-directed sites are potential substrates of GSK-3β protein kinase, and may be subject to kinase-dependent regulation. Unlike pT217, pS214 was not elevated in the CSF compared to the brain. This extracellular enrichment of pT217 over pS214 agreed with kinetic differences we previously identified within cells for these isoforms (Sato et al., 2018), with pT217 having a shorter turnover rate than unphosphorylated tau and tau-pS214.

Alternatively, decreased phosphorylation observed on some of the tau sites in brain extract compared to CSF could also result from partial dephosphorylation occurring during the PMI (Matsuo et al., 1994). Though such a decrease was not observed within the 5–16 h PMI range, modification of phosphorylation ratios during the PMI cannot be excluded. Assessing the kinetics of dephosphorylation of these tau sites from brain biopsies by MS would help to address the impact of this phenomenon on CSF and brain comparisons in the future.

Conversely, pS202 was significantly lower in the CSF and pS199 and pS202 were not elevated in AD, indicating that these phosphorylations may promote tau sequestration inside neurons. T181 is equally phosphorylated in the CSF and the brain. pT205 and pS208 were exclusively detected in CSF tau and not in soluble tau protein in the brain. This is interesting considering that the triple phosphorylation at S202, T205 and S208 is recognized by the anti-AT8 antibody (Malia et al., 2016) commonly used to characterize Braak stages of tau aggregation in brain (Braak and Braak, 1995). Indeed, pS208 was detected by MS in PHF (Hanger et al., 1998). pT205 and pS208 were absent in normal soluble brain tau in our study, confirming the unlikeliness to detect immunoreactivity of AT8 in normal brain tau. Furthermore, a recent study implicates pT205 and pS208 as a combinational phosphorylation pattern that, together with pS202, leads to tau self-aggregation (Despres et al., 2017). Exclusive presence of pT205 and pS208 in the CSF and further increased rates of phosphorylation at both sites in AD could indicate a potential protective clearance mechanism for neurons to remove these pathology-prone p-tau species from the cells. Simultaneous decrease of pS202 in AD CSF could also correspond to an increased sequestration of associated isoforms when aggregated with pT205 and pS208 in enriched AT8-positive tangles in the AD brain. Alternatively, absence of phosphorylation on pT205 and pS208 in brain extracts could result from their specific degradation by phosphatases occurring during the PMI. The rapid disappearance (<3 h) of AT8 reactivity reported on tau, collected from brain biopsy, supports this idea (Matsuo et al., 1994). Thus, such phosphatase activity efficiently targeting pT205 and pS208 could also be seen as an additional mechanism causing short a half-life of AT8-positive material in neurons. The increase of pT205 and pS208 found in AD CSF would then indicate potentially pathological decreasing of this protective mechanism. Finally, simultaneous decrease of pS202 in AD CSF could also correspond to an increased sequestration of associated isoforms when aggregated with pT205 and pS208 in enriched AT8-positive tangles in the AD brain.

We were unable to provide convincing detection of phosphorylated residues from the MTBR domain in the soluble fraction from normal brain. Phosphorylation sites on the MTBR domain have been purported to reduce the affinity of tau to microtubules (Biernat et al., 1993). The absence of such phosphorylation in normal tau could support the abnormality of these modifications found in PHF (Hanger et al., 2007). CSF tau truncation restricts the examination of phosphorylation changes *in vivo* since the MTBR domain likely degraded within the cell after tau truncation (Kanmert et al., 2015), and so was not recovered by the immuno-capture method used in this study.

### CSF p-tau Rates as AD Biomarkers

In addition to the significantly increased number of phosphorylation sites detected, we demonstrated how tau phosphorylation rate or stoichiometry can be quantified independent of global tau concentration. This concept is frequently overlooked but critical in assessing CSF tau phosphorylation changes in AD when absolute CSF p-tau concentration may increase solely due to increased t-tau concentration (i.e., pT181), and not due to an increase in the relative phosphorylation rate itself. Phosphorylation rate measurements from this study enabled for the first time the comparison of the degree and distribution of phosphorylation rate changes (hyper- vs. hypo-phosphorylation) across the protein, in different compartments (intracellular brain vs. extracellular CSF), and in different pathological conditions (AD vs. non-AD). This method could be used in the future to look at modifications of AD brain phosphorylations on numerous sites across brain regions and Braak stages as recently performed using immunochemistry (Neddens et al., 2018).

In this study, we demonstrated that tau in AD CSF is generally hyperphosphorylated in comparison to non-AD CSF. However, the degree of hyperphosphorylation is site dependent. T111, T205, S208 and T217 were more hyperphosphorylated than T181, which is the most commonly measured target used as a p-tau biomarker for AD (Fagan et al., 2009). We have also identified T153 phosphorylation that was exclusively found in AD CSF. Interestingly, the sites found hyperphosphorylated in AD CSF correspond to the sites already significantly increased in normal CSF compared to the brain (ie T111, T205, S208, and T217, and to a lesser extent T231). This would indicate AD pathology exacerbates cellular mechanisms contributing to specific p-tau isoforms enrichment during tau release into the CSF. Overall, the high magnitude of change found on these sites lets us envision their use as sensitive biomarkers to detect AD. Our study relied on a limited number of CSF pools and future studies on larger CSF cohorts would better establish potential relationships between p-tau changes and brain amyloidosis and tau aggregation over the course of the disease. We can also ask whether there are any specific p-tau profile changes in other non-AD tauopathies such as PSP, CBD, and FTD. Alternatively, this method could be used to track different kinase activities *in vivo* and *in vitro* and may also provide a promising tool to assess new drugs targeting abnormal tau metabolism.

## Author Contributions

NB conceived the project and designed the study. NB, CS, PM, and NM performed experiments, analyzed the data, and interpreted the data. NB, CS, NM, and RB wrote the article.

## Conflict of Interest Statement

The authors declare that the research was conducted in the absence of any commercial or financial relationships that could be construed as a potential conflict of interest.
